# Assessment of Service Delivery Modifications by Providers Serving Families With Developmental Disabilities During the COVID-19 Pandemic

**DOI:** 10.7759/cureus.84738

**Published:** 2025-05-24

**Authors:** Hannah Kwak, Nicole M Jacobs, Denise M Nunez, Priyanka Fernandes

**Affiliations:** 1 Department of Medicine, University of California, Los Angeles David Geffen School of Medicine, Los Angeles, USA; 2 Department of Pediatrics, California University of Science and Medicine, Colton, USA

**Keywords:** best practices, covid-19, developmental disabilities, modified delphi, service delivery

## Abstract

Background and objective

The COVID-19 pandemic has forced healthcare professionals to find innovative ways to deliver healthcare services. The objective of this study is to understand service modifications made by providers to continue care for children with developmental disabilities (DD) and their families during the height of the COVID-19 pandemic and develop best practices for future emergencies.

Methods

A modified Delphi method was used to understand service delivery modifications for children with DD and providers’ perspectives on these modifications. Ten providers from multiple specialties rated 16 modified practices during the COVID-19 pandemic on four measures: frequency of use in their individual practice, helpfulness in delivering care and/or meeting clients’ learning needs, feasibility of delivering care and/or meeting clients’ learning needs, and likelihood to use after the COVID-19 pandemic. A three-round modified Delphi process with an expert panel discussion was conducted to achieve consensus agreement on best practices.

Results

Consensus, defined as at least 70% of the panelists giving a high rating (4 or 5) or low rating (1 or 2) for a modified practice, was reached for a total of nine (56%) of the 16 modified practices. Panelists agreed that modifications helpful for service delivery beyond the pandemic included dedicated staff for follow-up and referrals; electronic portals to send reminders, information, and handouts; personalized handouts and materials targeting clients’ needs; and real-time client feedback. Panelists also reached a positive consensus that virtual visits, alternate service delivery settings, and the provision of personal protective equipment were helpful and feasible to enhance care delivery. However, negative consensus was noted on keeping in-person visits brief and strictly for essential activities.

Conclusions

This study highlights important service modifications that were helpful during the COVID-19 pandemic from the perspective of service providers.

## Introduction

Children with developmental disabilities (DD) encompass a wide range of conditions, including autism spectrum disorder, Down syndrome, fragile X syndrome, cerebral palsy, and fetal alcohol syndrome. These children with DD and their caregivers/families often access crucial services through the healthcare and/or educational settings to support their diverse set of needs. In the best of times, the system of services can be fragmented and difficult to navigate [1]. In the event of unexpected and prolonged disruptions to the system, as during the SARS-CoV-2 strain of the COVID-19 pandemic, children, caregivers, and families can quickly become burdened and overwhelmed in achieving the same independence and health goals [2].

Youth with DD have been especially vulnerable to disrupted care during the pandemic due to their social, emotional, and mental vulnerabilities that have translated to greater healthcare needs, mental health concerns, and dependency on services [3,4]. Disproportionately high levels of stress during the pandemic were associated with a decreased quality of life in these populations [5]. Studies have shown that most parents of children with DD reported having to devote extra time at home to address a lack of services provided to their children as a result of the COVID-19 pandemic, and the majority of parents reported that this lack of services negatively impacted their child’s social and educational development [6-8].

The COVID-19 pandemic has forced healthcare professionals to find innovative ways to deliver healthcare services while complying with public health guidelines and preventing the spread of the virus. In particular, there has been a rapid rise in the use of online platforms to deliver healthcare services [9]. Recent data has shown that telehealth advancements have helped providers improve service outreach and resources to people who are geographically isolated or have transportation difficulties [10]. However, researchers found that over 70% of individuals with DD experienced some loss of health services due to COVID-19 despite the use of telehealth advancements [11]. Even with technological advancements, some individuals with DD do not have adequate electronic substitutions for remote therapy, education, and interaction, making disruptions to care even more of an urgent concern for this population [9]. Individuals with DD expressed that they lost at least some aspect of their critical care services during the COVID-19 pandemic [11]. Specifically, researchers have found that 56% of individuals with disabilities surveyed reported that access to regular health care treatment was disrupted during the pandemic, and 23% of respondents who regularly used a direct care worker were no longer receiving these care worker services during the COVID-19 pandemic [11]. Of note, there was an alarming decrease in the number of individualized education programs and services during the COVID-19 pandemic, including a reduction in access to occupational and physical therapy [12].

The current understanding of providers’ perspectives on supporting individuals with DD in the setting of large disruptions like the COVID-19 pandemic is limited, and there has yet to be a systemic consensus established on which service modifications to maintain after such emergency disruptions. Providers caring for individuals with DD in an independent living setting during the COVID-19 pandemic have highlighted important considerations while serving this population, including continued management of chronic health conditions, increasing use of personal protective equipment (PPE) in congregate living homes, addressing social isolation for an already vulnerable population, and pursuing recertification of governmental economic support [13]. Adaptive practices adopted during emergencies can be difficult to systematically study in terms of feasibility and effectiveness but are important to document and include as best practices in provider preparedness planning for future emergencies.

The aim of this study was to understand the variety of service delivery modifications made by providers to support children with DD and their families during the height of the COVID-19 pandemic, prior to widespread availability of preventive and treatment options. Furthermore, the goal of this study was to develop a set of best practices in service delivery for provider consideration in preparing for future pandemics and emergencies. To achieve this, the study leveraged a framework to elicit best practices in the setting of limited evidence using the modified Delphi technique, a process by which experts in the field were surveyed and reached a consensus decision on service modifications during the pandemic.

## Materials and methods

Study design

The modified Delphi method is a consensus-building approach among experts to help establish guidelines or quality indicators on topics that have little available definitive evidence. It is useful when statistical models are not readily available, when human expertise plays a role in understanding the research question, and when there are recent developments in the problem that make our knowledge incomplete [14,15]. The method uses an iterative process of expert rating and panel discussion in creating a consensus and is often the preferred method in clinical practice guideline development [15].

**Figure 1:**
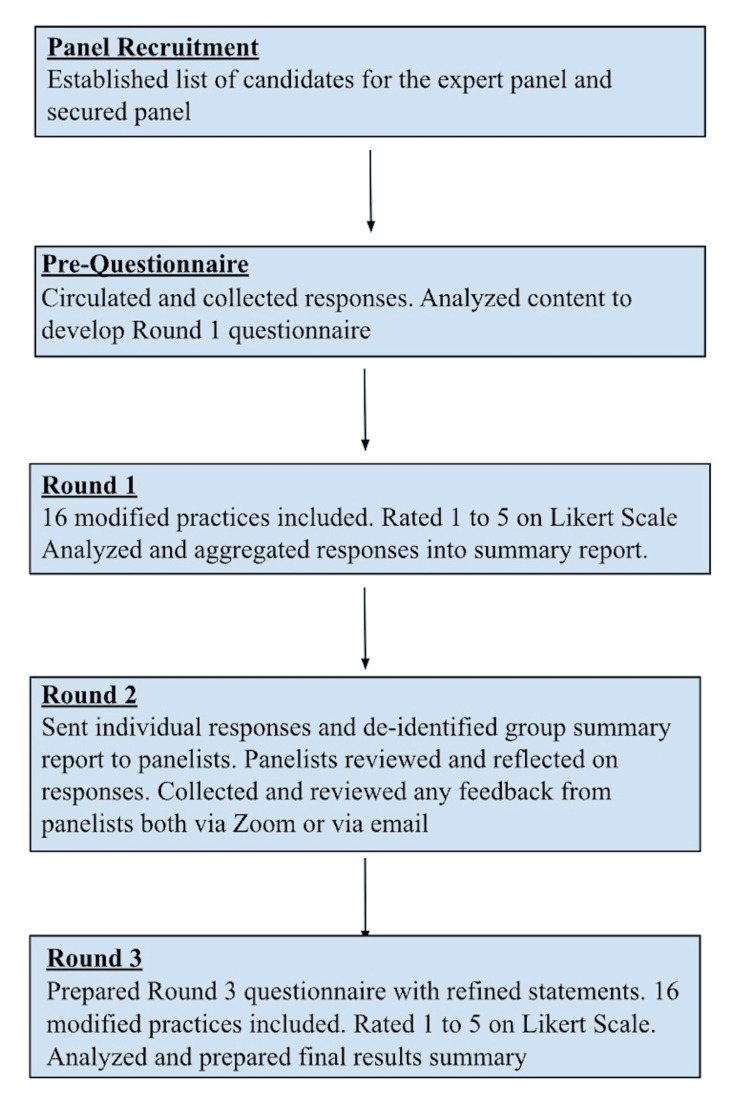
Overview of the modified Delphi process used in the study

The study was conducted between January and September 2021, at the height of the COVID-19 pandemic and prior to the widespread availability and use of vaccines and medications. There were no previously described or proven strategies available at the time to guide clinical/educational service providers’ decisions to use alternate delivery strategies such as telehealth, outdoor service delivery, etc. The study team therefore used the modified Delphi method to understand providers’ perspectives on the benefits, barriers, and feasibility of service delivery adaptations used for children with DD. The consensus process incorporated an open-ended questionnaire followed by a three-round modified Delphi process (Figure 1). In following infection prevention protocols and facilitating engagement during the pandemic, all surveys and expert panel discussions were conducted remotely. Surveys were all administered via the REDCap platform, and the panel discussion was held on the Zoom platform. Panelists were provided $25 gift cards for every round of participation as incentives to improve participation and engagement throughout the study period. The University of California, Los Angeles Institutional Review Board (FWA00004642) approved the research project on July 19, 2022 and renewed its approval on July 19, 2023, as it met requirements for expedited review without the need for continuing review per 45 CFR 46.110 Category 7.

Panel selection

The study team recognized the variety of clinical and educational service delivery settings and the continuously changing needs of children with DD during the pandemic. A heterogeneous panel of experts was sought because service delivery adaptations in a particular specialty may have been underutilized in other settings and could potentially be considered for future use in those settings. Prior analyses have suggested that having a minimum of seven panel members ensured diversity while allowing for all members to be involved in discussions/rankings [16]. To account for potential participant “no-shows,” the panel size recruited 10 members to ensure adequate disciplinary representation and study retention while providers faced busy schedules throughout the COVID-19 pandemic. Panel member nominations were solicited from clinicians and providers working at the University of California Leadership Education in NeuroDiversity (UC-LEND) clinic. The UC-LEND is a comprehensive, multidisciplinary clinic that specializes in caring for individuals with neurodevelopmental disabilities and their families. In keeping the practice recommendations developed through the study current and easily translatable to the clinical and educational setting, panel members who were actively providing services before and during the pandemic were recruited. Panel members were sent email invitations outlining the goals/process of the study, and consent was obtained via email. The final panel included specialists in child development, pediatrics, occupational therapy, neurology, high school special education counseling, speech-language pathology, social work, and case management (Table 1).

**Table 1:** Characteristics of the modified Delphi expert panelists

Characteristic	n (%)
Total	10
Specialty service	
Child development	3 (30%)
Pediatrics	1 (10%)
High school counselor – special education	1 (10%)
Speech and language pathology	1 (10%)
Occupational therapy	1 (10%)
Social work and welfare	1 (10%)
Case management	1 (10%)
Neurology – behavioral child	1 (10%)
Gender	
Male	1 (10%)
Female	8 (80%)
Choose not to disclose	1 (10%)
Years of experience in specialty service	
Less than 5	3 (30%)
5-10	2 (20%)
10-15	3 (30%)
More than 15	2 (20%)
Employer	
Hospital/clinic-based	4 (40%)
Third-party service provider	1 (10%)
Public school	1 (10%)
Private school	0 (0%)
Other	4 (40%)
Race/ethnicity	
White American	2 (20%)
Black or African American	0 (0%)
Native American or Alaska Native	0 (0%)
Asian American, Native Hawaiian, or Pacific Islander	5 (50%)
Latinx	2 (20%)
I do not wish to answer	1 (10%)

Evidence development

In identifying service delivery modifications that were previously tested and recommended, the lead author of the study team conducted a literature review prior to the start of the consensus development. The review elicited the benefits and challenges of telehealth practices in providing services to children with DD and their families, but there was a dearth of information on other service delivery modifications. The study team therefore developed an open-ended questionnaire to identify a more comprehensive list of service delivery modifications that were being used by the panel members as front-line providers during the pandemic. The questionnaire (Appendix A) requested basic demographic information and a list of adaptations made to service delivery for children with DD (referred to as clients in the questionnaire) compared to before the pandemic. Surveys were distributed via the REDCap platform. The response rate was 8 (80%). The lead author analyzed survey responses to identify similar terms and modified practices and grouped them into common themes. The analysis resulted in 16 modified practices and four themes (Table 2). The list of modified practices was reviewed for appropriateness and relevance by two non-study clinical researchers from UC-LEND. Based on recommendations received, we included all 16 modified practices in the consensus development.

**Table 2:** List of modified practices included in round 1 and round 3 surveys PPE, personal protective equipment

Modified practices included in the round 1 and round 3 surveys
Administrative modifications
Dedicated staff responsible for assisting with follow-up and navigating referral services
Dedicated staff responsible for troubleshooting technological issues prior to remote visits
Keeping in-person visits brief and strictly for essential activities only
Increase hours available to provide services
Increase the days available to provide services
Use existing secure electronic client-provider portals to send clients reminders, information, and handouts
Use external secure platforms to send clients information and handouts
Personalizing online handouts and materials targeting clients’ needs
Service setting modifications
In-person visits in outdoor spaces
Virtual telephone/audio-only visits
Virtual audio and video visits
Infection prevention modifications
COVID-19 pre-screening protocols for in-person visits
Provide PPE to providers for in-person visits
Provide PPE to clients for in-person visits
Use of real-time satisfaction data
Obtain client feedback to improve and/or tailor service delivery
Provide feedback to the administration on modified service delivery to improve and/or tailor service delivery

Consensus development

In round 1, panel members were asked to rate each of the 16 modified practices on four measures: frequency of use in their individual practice, helpfulness in delivering care and/or meeting clients’ learning needs, feasibility of delivering care and/or meeting clients’ learning needs, and likelihood of using the service after the COVID-19 pandemic. The rating was a 5-point Likert scale, where 1 was “not at all” and 5 was “extremely.”

Panelists were given two weeks to complete the survey, and reminders were sent one week and one day prior to the submission deadline [17,18]. In round 2, the study team organized a virtual panel discussion using the Zoom platform. However, due to scheduling difficulties and demanding workloads of providers during the pandemic, only two of the 10 panelists participated in the panel discussion. The discussion was moderated by two members of the study team. To potentially reduce the level of disagreement between panel members, emails were sent to each panel member with their personal ratings from round 1 and a de-identified, aggregate report of ratings from other panelists from round 1. Panelists were asked to review these results over two weeks, with a reminder email set up at one week and one day prior to sending the questionnaire for round 3. During the two-week period, email communication between panel members was allowed and encouraged to foster discussion and clarification between panel members and the study team that was not adequately achieved due to the low attendance of the virtual discussion. Although the expert panel discussion was aimed at facilitating an increase in consensus, it was noted that through this modified process, there was an improvement in consensus regarding some modified practices in round 3.

In round 3, panel members were asked to complete the same questionnaire from round 1 after reflecting on their personal responses and group responses. As they reported minimal confusion, comments, or clarification with the questionnaire, no revisions were made to the round 1 survey for round 3. Panelists were given two weeks to complete the survey, with a reminder sent one week and one day prior to the submission deadline.

Data analysis 

For this study, consensus was achieved a priori when at least 70% of the panelists gave a high rating (4 or 5) or low rating (1 or 2) for a modified practice [19-21]. A consensus rating of 4 or 5 suggested that the panel frequently used or found a modified practice to be helpful, feasible, or likely to continue after the pandemic, also defined as a positive consensus. On the contrary, a 70% or greater consensus rating of 1 or 2 suggested that the panel infrequently used a modified practice or found it unhelpful, not feasible, or unlikely to continue, and thus was defined as a negative consensus. Panelist responses were analyzed by members of the study team, and a summary of responses that did and did not meet consensus was compiled for both rounds 1 and 3.

## Results

A total of 10 healthcare professionals consented to participate in the study. As shown in Table 1, experts had a range of experiences and were from varied specialties. Of the participating experts, seven identified as having at least five years of experience providing services to youth with DD and their families, with the median years of service being 9.5 years. Most panelists practiced in Los Angeles County; only one practiced in Riverside County. The response rate was 8 (80%) for round 1 and 5 (50%) for round 3. At the end of round 3, consensus was reached for a total of nine (56%) of the 16 listed modified practices. With respect to the four measures of practice efficacy within each modified practice, consensus was reached 24 (37%) out of a potential 64 times. Outlined below is the summary of the results, organized by the four themes: administrative modifications, service setting modifications, infection prevention modifications, and use of real-time satisfaction data.

Administrative modifications

Modified practices in this theme included having dedicated staff assisting with follow-up of and navigation of referral services, having dedicated staff responsible for troubleshooting technological issues prior to remote visits, keeping in-person visits brief and strictly for essential activities only (e.g., hearing screen, vision screen, etc.), increasing hours available to provide services, increasing days available to provide services, using existing secure electronic client-provider portals to send clients reminders, information and handouts, using external secure platforms to send clients information and handouts, and personalizing online handouts and materials targeting clients’ needs (Table 2). Positive consensus was reached for having dedicated staff assisting with follow-up and navigation of referral services, providing additional days of service delivery, using existing secure electronic client-provider portals to send clients reminders, information, and handouts, and personalizing online handouts and materials targeting clients’ needs (Table 3). Negative consensus was reached for keeping in-person visits brief and strictly for essential activities only (Table 4).

**Table 3:** Modified practice comparison of measures that reached positive consensus The asterisk (*) signifies statistics that are consistent with positive consensus. PPE, personal protective equipment

Modified practices	Average Likert rating of measures			
	Frequency	Helpful	Feasible	Likely to continue
Administrative modifications				
Dedicated staff for follow-up/referrals	4 (80%)*	5 (100%)*	4 (80%)*	5 (100%)*
Dedicated staff for tech problems	1 (20%)	2 (40%)	2 (40%)	1 (20%)
Extended hours	3 (60%)	3 (60%)	2 (40%)	0 (0%)
Additional days	1 (20%)	4 (80%)*	2 (40%)	0 (0%)
Virtual reminders/info/handouts	5 (100%)*	4 (80%)*	4 (80%)*	5 (100%)*
Separate secure online platform for reminders/info	2 (40%)	1 (20%)	1 (20%)	0 (0%)
Personalized handouts	5 (100%)*	5 (100%)*	5 (100%)*	5 (100%)*
Dedicated schedules for brief in-person visits	1 (20%)	1 (20%)	1 (20%)	1 (20%)
Service setting modifications				
In-person visits outdoors	0 (0%)	2 (40%)	2 (40%)	1 (20%)
Phone visits	1 (20%)	1 (20%)	2 (40%)	2 (40%)
Virtual video visits	4 (80%)*	4 (80%)*	4 (80%)*	2 (40%)
Infection prevention modifications				
COVID-19 pre-screening	3.75 (75%)*	3 (60%)	3 (60%)	2 (40%)
Adequate PPE for providers	5 (100%)*	4 (80%)*	4 (80%)*	2 (40%)
Adequate PPE for clients	3 (60%)	1 (20%)	1 (20%)	0 (0%)
Use of real-time satisfaction data				
Continuous client feedback	3 (60%)	4 (80%)*	3 (60%)	4 (80%)*
Continuous provider feedback	2 (40%)	3 (60%)	3 (60%)	3 (60%)

**Table 4:** Accommodative practice comparison of measures that reached negative consensus The asterisks (**) signify statistics that are consistent with negative consensus. PPE, personal protective equipment

Accommodative practice	Average of Likert rating of measures			
	Low frequency	Low helpfulness	Low feasibility	Unlikely to continue
Administrative modifications				
Dedicated staff for follow-up/referrals	1 (20%)	0 (0%)	0 (0%)	0 (0%)
Dedicated staff for tech problems	2 (40%)	1 (20%)	1 (20%)	3 (60%)
Extended hours	2 (40%)	1 (20%)	2 (40%)	3 (60%)
Additional days	2 (40%)	0 (0%)	1 (20%)	2 (40%)
Virtual reminders/info/handouts	0 (0%)	0 (0%)	0 (0%)	0 (0%)
Separate secure online platform for reminders/info	2 (40%)	2 (40%)	3 (60%)	3 (60%)
Personalized handouts	0 (0%)	0 (0%)	0 (0%)	0 (0%)
Dedicated schedules for brief in-person visits	4 (80%)**	3 (60%)	3 (60%)	4 (80%)**
Service setting modifications				
In-person visits outdoors	2 (40%)	2 (40%)	2 (40%)	3 (60%)
Phone visits	2 (40%)	2 (40%)	2 (40%)	3 (60%)
Virtual video visits	0 (0%)	0 (0%)	0 (0%)	1 (20%)
Infection prevention modifications				
COVID-19 pre-screening	1.25 (25%)	1 (20%)	1 (20%)	1 (20%)
Adequate PPE for providers	0 (0%)	0 (0%)	0 (0%)	1 (20%)
Adequate PPE for clients	1 (20%)	2 (40%)	1 (20%)	2 (40%)
Use of real-time satisfaction data				
Continuous client feedback	0 (0%)	1 (20%)	1 (20%)	0 (0%)
Continuous provider feedback	0 (0%)	0 (0%)	0 (0%)	0 (0%)

Positive panel consensus was met for all four measures of efficacy, including frequency of use, helpfulness, feasibility, and likelihood of future use for three of the four modified practices (i.e., having dedicated staff assisting with follow-up of and navigation of referral services, using existing secure electronic client provider portals to send clients reminders, information, and handouts, and personalizing online handouts and materials targeting clients’ needs) (Table 3). For the modified practice of providing additional days of service delivery, while the panel met consensus in terms of its helpfulness, it did not meet consensus in relation to the frequency of use, feasibility, and likelihood of its use in the future. Panelists reached a negative consensus for the frequency and likelihood of future use of keeping in-person visits brief and strictly for essential activities, reflecting that this practice was used infrequently and unlikely to be used in the future (Table 4).

Service setting modifications

Modified practices in this theme included conducting in-person visits in outdoor spaces, conducting virtual telephone/audio-only visits, and conducting virtual audio and video visits (Table 2). Positive consensus was met for conducting virtual audio and video visits (Table 3). Although positive consensus was met with respect to the frequency, helpfulness, and feasibility of conducting virtual audio and video visits, no consensus was reached on the likelihood of future use of this practice.

Infection prevention modifications

Modified practices within this theme included conducting COVID-19 pre-visit screening for in-person visits, providing PPE to providers for in-person visits, and providing PPE to clients for in-person visits (Table 2). Positive consensus was met for the modified practices of conducting COVID-19 pre-visit screening for in-person visits and providing PPE to providers for in-person visits. Of the four measures of efficacy, panelists reached consensus on the frequency of use of the practice of conducting COVID-19 pre-visit screening for in-person visits (Table 3). Panelists reached consensus in terms of frequency of use, helpfulness, and feasibility of providing PPE to providers for in-person visits, although they did not reach consensus in terms of the likelihood of future use.

Use of real-time satisfaction data

Modified practices within this theme included obtaining continuous client feedback and obtaining continuous provider feedback (Table 2). Positive consensus was met for the modified practice of obtaining continuous client feedback (Table 3). Panelists reached consensus in terms of the helpfulness of this practice and the likelihood of its future use, although they did not reach consensus in terms of the frequency of use and feasibility.

## Discussion

The current study was performed at a time when the threats from the pandemic were still significant, well before the widespread use of COVID-19 treatments and vaccines. Children with DD and their families were particularly burdened with the need to access health and educational services without knowing the best and safest way to do so. Individual providers and individual family units were therefore forced to provide and access services with minimal available evidence at the time. Our study highlights some important service modifications that were undertaken and found to be successful in the setting of the pandemic and from the perspective of service providers. The study highlights some modifications that our service providers were planning to use long term, suggesting an opportunity to create systems in service delivery that are more pandemic- or emergency-ready. Lastly, we highlight a rapid and systematic approach to develop best practices in service delivery in the absence of robust research and evidence through the modified Delphi process, a methodology that can be considered during future pandemics.

Of particular interest, our study showed the highest level of consensus for some of the administrative modifications that were made during the pandemic. Service providers noted that personalizing information to fulfill clients’ unique needs was not only helpful and feasible during the pandemic but could be of benefit even post-pandemic. Previous studies have shown that implementing individualized communication methods for both children and adults with disabilities can improve healthcare interactions, help limit the high levels of patient-provider dissatisfaction, and work to reduce the poorer healthcare outcomes associated with this community [22-24]. Providers in our study overwhelmingly agreed that providing children and families with dedicated administrative support before and after the provision of services in the form of appointment reminders, handouts, and referral navigation was helpful and feasible. The findings affirmed the existing realities of many families wherein services offered are fragmented, and having navigation services has often benefited families [25]. Adding additional days of service was noted to be helpful by most panelists, although there was no consensus on its feasibility and long-term use. In the face of growing health provider burnout, providing additional days of service must be balanced with maintaining the well-being of the workforce [26]. The administrative practice that elicited a negative consensus among panelists was the practice of keeping in-person visits brief and strictly for essential activities only. Panelists did not frequently use this practice or think it would be used in the future, which suggests an overall preference for in-person interactions between health and education providers and clients.

During the pandemic, there was a rise in families choosing to participate in “pandemic pods,” wherein families organized their children into smaller groups to receive some component of in-person teaching and to enhance social interaction [27]. Pandemic pods occurred in various settings: virtual, hybrid, in-person indoors, and in-person outdoors. Looking back, parents who participated in these pods reported their children being equally or more engaged in the educational content and better adjusted to the learning environment compared to before the pandemic [28]. However, the average weekly cost for families was about $300 per child, raising concerns about the affordability of this model. The report also highlighted that many pandemic pods were neither equipped nor effective in serving children with special needs. Panelists in our study did not frequently engage in outdoor in-person visits and also did not attain consensus on the usefulness and feasibility of this practice. The study team received some insight around this practice during round 2 of the study (i.e., panel discussion) from panelists who expressed concerns about the presence of distractions and the lack of safe/confined spaces in certain outdoor settings. On the contrary, video visits were extensively used, and panelists achieved consensus in terms of their feasibility and helpfulness. Interestingly, however, consensus was not reached for the long-term use of video visits. This lack of consensus in all measures could be due to the fact that access to virtual visits prior to March 2020 can influence the use of telemedicine during and after the COVID-19 pandemic, as physicians who did not use telemedicine prior to the pandemic were more likely to express dissatisfaction with virtual visit options during the pandemic [29].

The pandemic forced a rapid expansion of telehealth services, allowing little room for systematically studying the determinants of a successful telehealth service as well as subpopulations that might most benefit from these services long-term [30-32]. Uncertainty around insurance reimbursement of telehealth services post-pandemic could have also been a reason why consensus was not achieved among panelists for this topic.

With relation to infection prevention measures adopted by providers, the highest consensus was achieved for ensuring providers had adequate PPE. Panelists did not agree that the use of PPE in clients was helpful or feasible, underscoring the common issues that may arise in having children with DD use PPE (e.g., face masks) [33]. However, since the study was conducted, there have been several interventions that can be used to teach those with DD to better tolerate facial masks and coverings [33-35]. Using interventions to promote mask wearing among children with DD might be important to consider in pandemic preparedness, particularly for infections that are easily spread via the droplet and airborne route.

Lastly, collecting real-time satisfaction data from clients was found to be helpful to providers. Patient satisfaction data are commonly used in the healthcare setting to help determine the quality of care being provided [36]. Using a similar approach in educational and other health service delivery settings might be helpful to tailor practices to address the needs of patients. In a rapidly changing environment, such as the initial months of the pandemic, collecting real-time data also enables providers to ensure clients are abreast of changing public health recommendations. In contrast, there was no consensus opinion that provider feedback to administration was found to be as helpful or feasible in our study. This could be explained by the limited provider time available during the height of the pandemic to speak with administration and the strong emphasis on eliciting patients' experiences and feedback to drive administration-level changes for patient-centered care [37].

An aspect of the study that is important to highlight is the framework used to collect actionable data in an ever-changing environment. The study team adopted a multidisciplinary approach by engaging experts and providers from across specialties, researchers in developmental delays as well as in public health, self- and family advocates, and undergraduate and graduate students. Given that policies differ drastically between local jurisdictions, the team limited the geographic inclusion for the study (predominantly Los Angeles County) to keep recommendations easily translatable at a population level. The study was conducted over a period of nine months, which is a relatively short time frame to develop practice guidance for providers. It was possible because the team used a modified Delphi process, which allowed for the systematic collection of data at a time when public health recommendations were constantly changing and the evidence on best practices was unknown [14,15].

Overall, this study emphasizes the important service delivery modifications that were found to be helpful, feasible, used at a high frequency during the global pandemic, and likely to continue to be used after the pandemic by leveraging the modified Delphi technique to survey service providers when a set of best practices has yet to be determined.

Limitations

The biggest limitation of the study was the time frame during which it took place. It was the height of the COVID-19 pandemic, which limited the time panelists could devote to the multiple rounds of the modified Delphi method. In particular, round 2 of the modified Delphi process was poorly attended, limiting the opportunity for a robust discussion on the modified practices used by various providers in the field. As a workaround for this step of the process, a summary sheet was provided to all providers, allowing those who did not participate in round 2 an opportunity to look at group consensus opinions and review the reasoning behind other panelists’ selections. Panelists were given the opportunity to share their opinions via email instead. The team also ensured that panelists were incentivized to participate in each step of the study process. The study had a small sample size, which might prevent adequate representation of a large and heterogeneous population. The study team was intentional in including a heterogeneous group of providers and surveying them about past and future modified practices to implement. Lastly, the generalizability of results is somewhat limited due to the geographic limitations of the study participants.

## Conclusions

Overall, this study highlights important considerations that service providers can glean in terms of service delivery modifications in the setting of systemic disruptions that occurred during the COVID-19 pandemic. Panelists agreed that some modifications adopted during the pandemic, including having dedicated staff assisting with follow-up of referral services, using secure electronic client-provider portals to send clients reminders, information, and handouts, personalizing online handouts targeting clients’ needs, and using real-time client feedback, would be helpful to use even beyond the pandemic. Despite the unique considerations for clients with DD, the study also highlights how practices such as the use of virtual visits, alternate service delivery settings, and PPE were found to be helpful and feasible in the short term. Lastly, the study leveraged a framework to elicit best practices in the setting of limited evidence that can be used in future emergencies or pandemics. Future research can expand this work by investigating the regional differences between service providers during the pandemic and contrasting this to the preferences of service providers that do not work with clients with DD.

## Appendices

Appendix A: Survey questions experts' pre-questionnaire

**Table 5:** Survey questions experts pre-questionnaire The pre-questionnaire was distributed to all panelist members. Responses from the pre-questionnaire informed the 16 modified practices assessed in the Round 1 and Round 3 questionnaires.

Question	Answer Choices (blank for open-ended questions)
Where did you hear about the study?	Santa Monica LEND
	Riverside LEND
	UCLA primary care clinic
	UCLA neurology clinic
	Other
Are you of Latinx origin?	Yes
	No
	I do not wish to answer
Please indicate your ethnicity. (If answered “Yes” in question 2, question 3 shows up)	Mexican American
	Chicano/a/x
	Puerto Rican
	South American
	Other (please specify)
	I do not wish to answer
Please indicate your race. If two or more races, please check all that apply.	White American
	Black or African American
	Native American or Alaska Native
	Asian American, Native Hawaiian, or Pacific Islander
	I do not wish to answer
What language do you speak most often?	English
	Spanish
	Other (please specify)
What language do you feel comfortable using when providing services to patients/clients? Please check all that apply	English
	Spanish
	Other
Which of the following do you use to describe your gender identity?	Male
	Female
	Transgender male
	Transgender female
	Nonbinary
	I do not identify with any of the above
	Choose not to disclose
What is the highest degree you have obtained?	High school diploma or equivalency (GED)
	Associates degree (junior college)
	Bachelor’s degree
	Master’s degree
	Doctorate
	Professional (MD, JD, DDS, etc)
	Other
	None achieved before high school
What is your specialization of service?	
Select the description of your current employer	Hospital/clinic-based service provider
	Third party service provider
	School
	Public
	Private
	Regional center provider
	Other: specify
County of practice. Please check all the apply	LA county
	Riverside county
	San Bernardino county
	Other: specify
How many years of service do you have in your field of practice?	
What modifications have you made to your service delivery to accommodate patients/clients during the COVID-19 pandemic? (e.g. switching to remote services, in-person visits at original site with strict COVID-19 protocols)	
Of the modifications you listed above, which ones, if any, may you retain regardless of the COVID-19 pandemic? (e.g. keeping the option of virtual services)	
What modifications were most helpful for your patients to achieve their goals and try to have their needs met?	
What modifications did your patients find feasible?	
What modifications did you want to implement during the COVID-19 pandemic but were not able to?	
Of the modifications you listed above, which ones, if any, were you unable to implement due to finances?	
What additional barriers outside of the direct service delivery has impacted your ability to provide services? (e.g. patient does not have access to technology or quiet setting to receive services, patient does not have printer access)	
What additional facilitators outside of the direct service delivery has impacted your ability to provide services? (e.g. lack of staffing, technological limitations, coordination of providers) We plan to develop an interactive forum to support patients and their families.	
In your opinion, what would be the 3 essential steps that parents can take to ensure optimal learning for their children during a pandemic, such as COVID-19?	
In your opinion, what would be the 3 essential steps that service providers can take to ensure optimal learning for their clients/patients during a pandemic, such as COVID-19?	

Appendix B: Expert panel - questionnaire for round 1 and round 3

**Table 6:** Expert panel questionnaire for round 1 and round 3 This questionnaire was used for both Round 1 and Round 3 to assess and obtain consensus from panelist members on 16 modified practices that were identified in the pre-questionnaire. The same questionnaire was used for both rounds as panelists did not identify any changes to update the questionnaire from Round 1 to Round 3. Please rate the following on a scale from 1 to 5. Respective to the question, where: (1) not at all (2) slightly (3) somewhat (4) very (5) extremely.

Service Modification	Measures to Rate
1. Virtual video visits (e.g. Zoom, Google Meet, Google Hangouts)	How frequently do you use/do this in your practice?
	How helpful is this for delivering care and/or meeting clients’ learning needs?
	How feasible is this for delivering care and/or meeting clients’ learning needs?
	How likely will you use this service after the COVID-19 pandemic?
2. Telephone or virtual telephone visits (e.g. Google Voice)	How frequently do you use/do this in your practice?
	How helpful is this for delivering care and/or meeting clients’ learning needs?
	How feasible is this for delivering care and/or meeting clients’ learning needs?
	How likely will you use this service after the COVID-19 pandemic?
3. COVID-19 pre-screening protocols for in-person visits	How frequently do you use/do this in your practice?
	How helpful is this for delivering care and/or meeting clients’ learning needs?
	How feasible is this for delivering care and/or meeting clients’ learning needs?
	How likely will you use this service after the COVID-19 pandemic?
4. In-person visits in outdoor spaces	How frequently do you use/do this in your practice?
	How helpful is this for delivering care and/or meeting clients’ learning needs?
	How feasible is this for delivering care and/or meeting clients’ learning needs?
	How likely will you use this service after the COVID-19 pandemic?
5. Fast-track schedules for brief in-person visits (e.g. vision screen, hearing screen)	How frequently do you use/do this in your practice?
	How helpful is this for delivering care and/or meeting clients’ learning needs?
	How feasible is this for delivering care and/or meeting clients’ learning needs?
	How likely will you use this service after the COVID-19 pandemic?
6. Increase hours available to provide services (e.g. late evening appointments)	How frequently do you use/do this in your practice?
	How helpful is this for delivering care and/or meeting clients’ learning needs?
	How feasible is this for delivering care and/or meeting clients’ learning needs?
	How likely will you use this service after the COVID-19 pandemic?
7. Increase days available to provide services (e.g. weekend)	How frequently do you use/do this in your practice?
	How helpful is this for delivering care and/or meeting clients’ learning needs?
	How feasible is this for delivering care and/or meeting clients’ learning needs?
	How likely will you use this service after the COVID-19 pandemic?
8. Provide personal protective equipment to providers for in-person visits	How frequently do you use/do this in your practice?
	How helpful is this for delivering care and/or meeting clients’ learning needs?
	How feasible is this for delivering care and/or meeting clients’ learning needs?
	How likely will you use this service after the COVID-19 pandemic?
9. Provide personal protective equipment to clients for in-person visits	How frequently do you use/do this in your practice?
	How helpful is this for delivering care and/or meeting clients’ learning needs?
	How feasible is this for delivering care and/or meeting clients’ learning needs?
	How likely will you use this service after the COVID-19 pandemic?
10. Dedicated staff responsible for trouble s hooting technological issues prior to remote visits	How frequently do you use/do this in your practice?
	How helpful is this for delivering care and/or meeting clients’ learning needs?
	How feasible is this for delivering care and/or meeting clients’ learning needs?
	How likely will you use this service after the COVID-19 pandemic?
11. Dedicated staff responsible for assisting with follow-up and navigating referral services	How frequently do you use/do this in your practice?
	How helpful is this for delivering care and/or meeting clients’ learning needs?
	How feasible is this for delivering care and/or meeting clients’ learning needs?
	How likely will you use this service after the COVID-19 pandemic?
12. Send virtual reminders, information or handouts to clients (e.g. MyChart, email)	How frequently do you use/do this in your practice?
	How helpful is this for delivering care and/or meeting clients’ learning needs?
	How feasible is this for delivering care and/or meeting clients’ learning needs?
	How likely will you use this service after the COVID-19 pandemic?
13. Use other secure online platform to send information or handouts to clients (e.g. DocuSign, RightFax, Doximity)	How frequently do you use/do this in your practice?
	How helpful is this for delivering care and/or meeting clients’ learning needs?
	How feasible is this for delivering care and/or meeting clients’ learning needs?
	How likely will you use this service after the COVID-19 pandemic?
14. Use or edit online handouts/material for clients	How frequently do you use/do this in your practice?
	How helpful is this for delivering care and/or meeting clients’ learning needs?
	How feasible is this for delivering care and/or meeting clients’ learning needs?
	How likely will you use this service after the COVID-19 pandemic?
15. Obtain client feedback to improve and/or tailor service delivery	How frequently do you use/do this in your practice?
	How helpful is this for delivering care and/or meeting clients’ learning needs?
	How feasible is this for delivering care and/or meeting clients’ learning needs?
	How likely will you use this service after the COVID-19 pandemic?
16. Provide feedback to administration on modified service delivery to improve and/or tailor	How frequently do you use/do this in your practice?
	How helpful is this for delivering care and/or meeting clients’ learning needs?
	How feasible is this for delivering care and/or meeting clients’ learning needs?
	How likely will you use this service after the COVID-19 pandemic?
